# The Effect of Zinc Oxide Nanoparticles on the Quantitative and Qualitative Traits of *Scutellaria baicalensis* Georgi in In Vitro Culture

**DOI:** 10.3390/ijms26125836

**Published:** 2025-06-18

**Authors:** Anna Krzepiłko, Roman Prażak, Agata Święciło, Jacek Gawroński

**Affiliations:** 1 Faculty of Food Sciences and Biotechnology, University of Life Sciences in Lublin, Skromna 8, 20-704 Lublin, Poland; anna.krzepilko@up.lublin.pl; 2 Institute of Plant Genetics, Breeding and Biotechnology, University of Life Sciences in Lublin, Akademicka 15, 20-950 Lublin, Poland; jacek.gawronski@up.lublin.pl; 3 Department of Environmental Microbiology, University of Life Sciences in Lublin, Leszczyńskiego 7, 20-069 Lublin, Poland; agata.swiecilo@up.lublin.pl

**Keywords:** antioxidants, carotenoids, chlorophylls, minerals, ZnONPs, *Scutellaria baicalensis* Georgi, polyphenols, tissue cultures

## Abstract

Zinc oxide nanoparticles (ZnONPs) are increasingly used in agriculture to stimulate plant growth and development, including under in vitro culture conditions. However, there is limited data on the effects of ZnONPs on the micropropagation of *Scutellaria baicalensis* Georgi. The pharmacological properties of this species make it a valuable medicinal plant. In Poland, it does not occur naturally but is cultivated for the production of herbal material. In vitro micropropagation is an effective method for obtaining genetically uniform plantlets. The aim of this study was to evaluate the effects of various concentrations of ZnONPs on growth parameters and the content of mineral nutrients, phenolic compounds, antioxidants, and photosynthetic pigments in *Scutellaria baicalensis* cultured in vitro. Shoot tip explants were cultured on MS medium supplemented with 1.0 mg dm^−3^ BA and 0.1 mg dm^−3^ IBA, together with ZnONPs at concentrations of 0 (control), 10, 20, 30, and 40 mg dm^−3^. The results showed that ZnONPs at concentrations of 10–20 mg dm^−3^ had no statistically significant effect on shoot or root development or on fresh weight gain. However, higher concentrations (30 and 40 mg dm^−3^) had a significantly negative impact on the number and length of shoots and roots, as well as on biomass accumulation. ZnONPs at 10–20 mg dm^−3^ significantly increased the content of potassium, calcium, magnesium, iron, and zinc in regenerated multi-shoot plantlets. A strong positive correlation (r = 0.951) was observed between ZnONP concentration and zinc accumulation in the plantlets. The levels of manganese and copper were not significantly different from the control. Plantlets treated with 30–40 mg dm^−3^ ZnONPs had significantly lower levels of calcium, iron, manganese, and copper. Those grown at 30 mg dm^−3^ had the highest potassium and magnesium levels, while plantlets exposed to 40 mg dm^−3^ had the highest zinc content. The total phenolic content and antioxidant activity (measured using ABTS and DPPH assays) were significantly higher in ZnONP-treated plantlets compared to the control. In contrast, the levels of chlorophyll a, chlorophyll b, total chlorophyll (a + b), and carotenoids were significantly lower in plants treated with ZnONPs. A strong negative correlation was found between ZnONP concentration and photosynthetic pigment content, while the ZnONP concentration was positively correlated with total phenolic content and antioxidant activity (ABTS^+^ and DPPH).

## 1. Introduction

One of the most interesting medicinal plants worldwide is *Scutellaria baicalensis* Georgi, a perennial herbaceous species growing to 40–50 cm in height, belonging to the family *Lamiaceae* Lindl. Its pharmacological properties have been utilized in traditional medicine for thousands of years in China and neighboring countries. Under natural conditions, it is found in East Asia, including parts of Mongolia, China, Japan, and Russia (particularly around Lake Baikal), as well as in several European countries [1,2,3,4].

Although the whole plant can serve as herbal material, the root and rhizome are most commonly used. *S. baicalensis* is rich in biologically active compounds, particularly lipophilic flavonoids such as baicalin, baicalein, wogonoside, wogonin, scutellarein, oroxylin A, and skullcapflavone. The pharmacological activities of *S. baicalensis* have been well documented, including antimicrobial effects, anticancer activity, cardiovascular protection, benefits in autoimmune diseases, and anti-obesity properties [5,6,7].

*Scutellaria baicalensis* is also classified as an adaptogenic plant with anti-stress and mild sedative properties. It is used to support the treatment of insomnia, stress, and anxiety, as well as to alleviate depressive symptoms [5]. Clinically, it is most often used to treat conditions such as the common cold and cough [6,7]. It is also recommended for individuals with allergic disorders, asthma, diarrhea, and recurrent urinary tract infections.

The active compounds in *S. baicalensis* extracts exhibit antibacterial, antifungal, and antiviral properties through various mechanisms, including biocidal and biostatic effects, inhibition of bacterial enzyme activity and toxins, prevention of biofilm formation, and antioxidant activity [8]. Compounds such as baicalin and baicalein have also demonstrated potential therapeutic effects against COVID-19 [9]. Dietary supplements containing *S. baicalensis* extracts are widely available. The plant also plays an important role in animal feed production, enhancing growth and productivity in poultry, pigs, and cattle [10]. Moreover, *S. baicalensis* has potential applications in various industrial sectors. Extracts from this species are used in the finishing of silk textiles, imparting high UV absorption capacity [11], and attempts have been made to produce packaging materials enriched with *S. baicalensis* extracts to reduce the spread of viruses and pathogenic bacteria [12].

*Scutellaria baicalensis* plant material can be obtained by several methods, most commonly by field cultivation and in vitro propagation. Plantation cultivation ensures a continuous supply of plant material. However, plants derived from conventional cultivation often show considerable genetic variability, and seed propagation typically requires stratification. Therefore, genetically uniform plantlets derived from in vitro cultures are often used in plantations [1,2,3]. In vitro cultivation of *S. baicalensis* provides a uniform source of plant material rich in bioactive compounds [13]. Additionally, in vitro production is independent of geographic location and variable field conditions [13,14,15].

In in vitro cultures, nanoparticles are increasingly used to reduce microbial contamination, accelerate embryogenesis, organogenesis, and tissue and plant growth, enhance the production of secondary metabolites, and facilitate genetic transformation and gene delivery to plant cells and organelles. Commonly used nanoparticles in in vitro systems include metal and metal oxide nanoparticles such as silver, copper, titanium, zinc, cobalt, nickel, and iron, as well as carbon-based nanoparticles (e.g., graphene, carbon nanotubes) and nanosilica [16]. Numerous studies have confirmed the stimulatory effect of zinc oxide nanoparticles (ZnONPs) on in vitro plant cultures [16,17,18,19,20].

Zinc plays a key role in plant development. It is involved in chlorophyll synthesis, improves photosynthetic efficiency, enhances the uptake and transport of minerals, and participates in the biosynthesis of plant hormones—particularly auxins and gibberellins, thereby mitigating oxidative damage caused by abiotic stressors [21]. Zinc is a component of the prosthetic groups of numerous enzymes, including aldolases, dismutases, anhydrases, dehydrogenases, isomerases, peptidases, phosphatases, transphosphorylases, and RNA and DNA polymerases. It is essential for auxin synthesis and the metabolism of carbohydrates, proteins, and phosphorus compounds. Zinc also plays a crucial role in cell division and protein synthesis, as well as in DNA replication and transcription. In zinc-deficient plants, protein synthesis rates and overall protein content are significantly reduced [22].

ZnO nanoparticles may protect plants from various abiotic stresses, including heavy metals, drought, and salinity, by alleviating their negative effects [19,21,22,23,24,25]. The application of ZnONPs as a nano-fertilizer enhances crop yield and reduces disease incidence due to their broad-spectrum antifungal and antibacterial activities [26].

The aim of this study was to investigate the effect of zinc oxide nanocolloids (ZnONPs) on *Scutellaria baicalensis* Georgi plants cultured in vitro. The morphological characteristics of *S. baicalensis* plantlets propagated in vitro were analyzed. The contents of selected mineral elements (potassium, calcium, magnesium, iron, zinc, manganese, and copper), chlorophylls a and b, total chlorophyll (a + b), carotenoids, total phenolic compounds (TPC), and total antioxidant capacity (TAC) in the fresh plant biomass were determined. To the best of the authors’ knowledge, the effects of ZnONPs on the development of *S. baicalensis* Georgi in in vitro culture have not previously been studied.

## 2. Results

### 2.1. Analysis of Morphological Characteristics

Regeneration of *Scutellaria baicalensis* Georgi explants was observed in all experimental variants, including the control and treatments with ZnONPs, resulting in the formation of plantlets with developed shoots and roots. The composition of the control medium effectively induced micropropagation. The highest average numbers of shoots and roots, as well as their greatest lengths, were recorded in the control group.

The addition of ZnONPs negatively affected the morphological parameters of *S. baicalensis* plantlets in comparison to the control. For ZnONPs concentrations of 10 and 20 mg dm^−3^, the number and length of shoots and roots were lower than in the control; however, these differences were not statistically significant. In contrast, statistically significant differences were observed at ZnONPs concentrations of 30 and 40 mg dm^−3^ (Table 1).

**Scheme 1:**
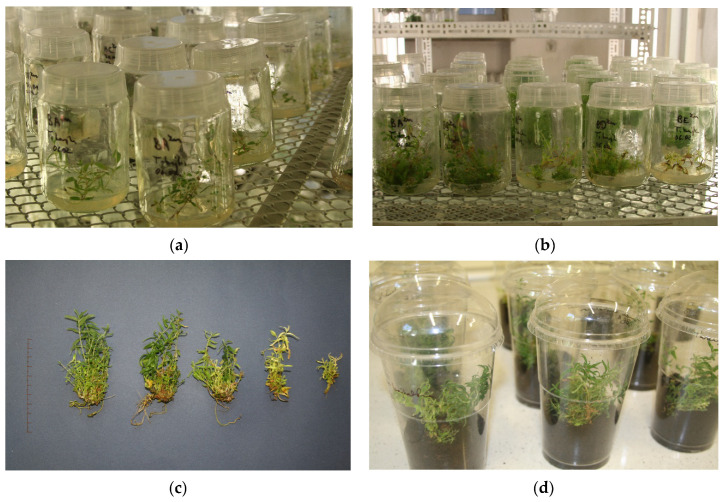
The influence of different ZnONPs treatments on the growth and development of *Scutellaria baicalensis* Georgi: (**a**,**b**) developing explants, (**c**) regenerated plantlets after three months: (from left) 0 (control), 10, 20, 30 and 40 mg dm^−3^ in MS medium, (**d**) regenerated plantlets in plastic cups filled with sterile soil.

The average number of *Scutellaria baicalensis* shoots in the treatments with 30 and 40 mg·dm^−3^ ZnONPs was lower than in the control by 44.8% and 72%, respectively. Similarly, for the same concentrations, the number of roots was 54.2% and 63% lower. The addition of ZnONPs at these highest concentrations also affected the length of the plant organs: shoot length was reduced by 34.6% and 65.8%, and root length by 50.6% and 77.8% for 30 and 40 mg·dm^−3^ ZnONPs, respectively. The highest fresh weights (0.686 g and 0.702 g) and fresh weight increments (0.657 g and 0.681 g) of micropropagated plantlets were recorded in the control and in the treatment with 10 mg·dm^−3^ ZnONPs (these values were not statistically significant). In the other experimental variants, as the concentration of ZnONPs increased, the fresh weight and fresh weight increment decreased compared to the control. In the treatments with 30 and 40 mg·dm^−3^ ZnONPs, the average fresh weight was 39% and 84% lower, respectively, than in the control, and the increase in fresh weight of the micropropagated plantlets was approximately 1.5 times and 6 times lower than in the control (Table 1, Scheme 1).

### 2.2. Evaluation of Mineral Content in Scutellaria Baicalensis Plant Material

Zinc oxide nanoparticles (ZnONPs) influenced the mineral composition of micropropagated *Scutellaria baicalensis* plantlets (Table 2). As the concentration of ZnONPs in the medium increased, there was a corresponding increase in the zinc content in the plants. The increase in Zn content ranged from 290% at a concentration of 10 mg·dm^−3^ to 1616% at 40 mg·dm^−3^ compared to the control. The in vitro control samples differed significantly in the content of K, Ca, Mg, Fe, and Zn from samples grown in the presence of ZnONPs. However, significant differences for Mn and Cu were only recorded at concentrations of 30 and 40 mg·dm^−3^ ZnONPs (Table 2). For concentrations of 10 and 20 mg·dm^−3^ ZnONPs, the content of K, Ca, Mg, Fe, and Mn was increased relative to the control, while the Cu concentration was slightly lower. The highest concentrations of Ca, Fe, Mn, and Cu were recorded for plants grown on media supplemented with 20 mg·dm^−3^ ZnONPs. The concentrations of K and Mg in micropropagated plantlets increased in the treatments with 10–30 mg·dm^−3^ ZnONPs in the medium, reaching their highest levels in the presence of 30 mg·dm^−3^ ZnONPs. The addition of 40 mg·dm^−3^ ZnONPs significantly reduced the concentrations of Mg, Fe, Mn, and Cu, which were approximately 16% lower, while the concentration of Ca was 12% lower and that of K was only about 3% lower than in the control (Table 2).

A positive Pearson’s correlation (r = 0.951) between the concentration of ZnONPs and the concentration of elements in *Scutellaria baicalensis* plantlets was observed only for zinc. In the control sample without zinc oxide nanocolloids, the concentrations of elements in the plantlets were ordered as follows: K > Ca > Mg > Fe > Mn > Zn > Cu. In plantlets from the 10 mg dm^−3^ ZnONPs treatment, the zinc content exceeded that of manganese; in the 20 and 30 mg dm^−3^ treatments, zinc content was higher than that of iron; and in the 40 mg dm^−3^ treatment, zinc content exceeded that of magnesium (Table 2).

The relative proportions of Cu, Zn, Mn, Fe, Mg, Ca, and K in the control plantlets were 1:19:33:50:231:671:3026. The addition of zinc oxide nanoparticles significantly increased the relative proportions of the minerals (Table 3).

### 2.3. Assessment of Total Phenolic Content in Extracts

**Figure 1:**
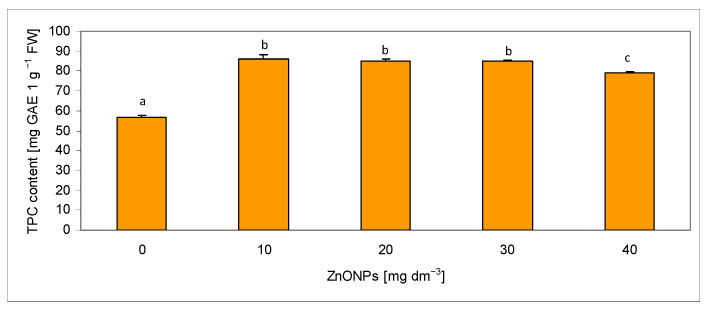
Total phenolic content (TPC) in fresh weight methanol extract of *Scutellaria baicalensis* Georgi plantlets. ZnONPs treatments: 0 (control), 10, 20, 30, 40 mg dm^−3^. Error bars represent ± SD. Different lowercase letters indicate significant differences at *p* < 0.05.

The total phenolic content (TPC) in methanolic extracts from the fresh biomass of *Scutellaria baicalensis* Georgi plantlets ranged from 56.58 mg GAE g^−1^ FW (control) to 85.85 mg GAE g^−1^ FW (at 10 mg dm^−3^ ZnONPs) (Figure 1). The TPC in all samples treated with zinc nanoparticles was significantly higher, by approximately 40–52%, than in the control. The Pearson correlation coefficient between ZnONPs concentration and TPC was positive (r = 0.631). The highest phenolic content was recorded in plantlets treated with 10 mg dm^−3^ ZnONPs, although it did not differ significantly from those treated with 20 and 30 mg dm^−3^ ZnONPs (85.24 mg GAE g^−1^ FW and 84.93 mg GAE g^−1^ FW, respectively). However, a decrease in TPC was observed in plantlets grown in the medium containing 40 mg dm^−3^ ZnONPs compared to those treated with lower nanoparticle concentrations (Figure 1).

### 2.4. Assessment of Total Antioxidant Capacity in Extracts

**Figure 2:**
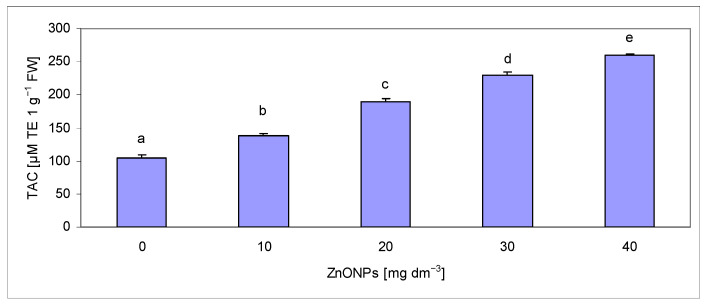
Total antioxidant capacity (TAC) in fresh weight of *Scutellaria baicalensis* Georgi plantlets determined by the ABTS method. ZnONPs treatments: 0 (control), 10, 20, 30, 40 mg dm^−3^. Error bars represent ± SD. Different lowercase letters indicate significant differences at *p* < 0.05.

The total antioxidant capacity (TAC) was measured in extracts from *Scutellaria baicalensis* plantlets using two methods based on the reduction of synthetic radical species, DPPH and ABTS^+^, and expressed as Trolox equivalent. Using the ABTS method, TAC values ranged from 104.77 to 259.31 μM TE g^−1^ FW (Figure 2). The addition of zinc oxide nanoparticles (ZnONPs) led to an increase in TAC values with rising nanoparticle concentrations, ranging from 32% to 147% compared to the control. In all experimental variants with ZnONPs, the total antioxidant capacity in the fresh mass of *S. baicalensis*, determined by the ABTS method, was statistically significantly higher than in the control. The highest TAC value was recorded in plantlets grown on medium supplemented with ZnONPs at a concentration of 40 mg dm^−3^. The Pearson correlation coefficient (r) between the ZnONP concentration and TAC measured by the ABTS method was r = 0.896 (Table 4).

**Figure 3:**
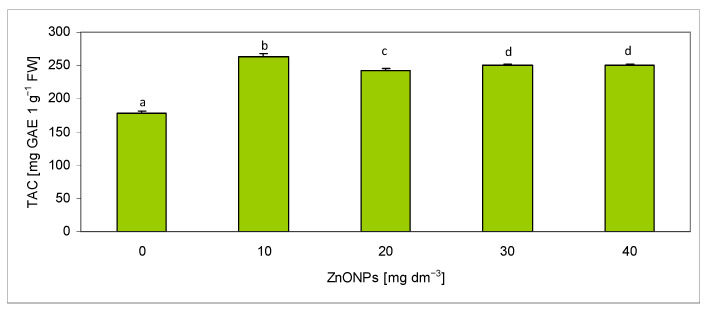
Total antioxidant capacity (TAC) in fresh weight of *Scutellaria baicalensis* Georgi plantlets determined by the DPPH method. ZnONP treatments: 0 (control), 10, 20, 30, 40 mg dm^−3^. Error bars represent ± SD. Different lowercase letters indicate significant differences at *p* < 0.05.

TAC determined using the DPPH method ranged from 178.84 to 262.82 μM TE g^−1^ FW (Figure 3). In all treatments with ZnONPs, the TAC value was statistically significantly higher, by 35–46%, than in the control. The highest TAC was observed at a ZnONPs concentration of 10 mg dm^−3^, representing a 46% increase relative to the control. At concentrations of 20, 30, and 40 mg dm^−3^, a slight but statistically significant decrease in antioxidant content was noted compared to the value measured at 10 mg dm^−3^ (Figure 3). The Pearson correlation coefficient (r) between ZnONP concentration and TAC determined by the DPPH method was r = 0.625 (Table 4).

### 2.5. Determination of Photosynthetic Pigments Content

**Figure 4:**
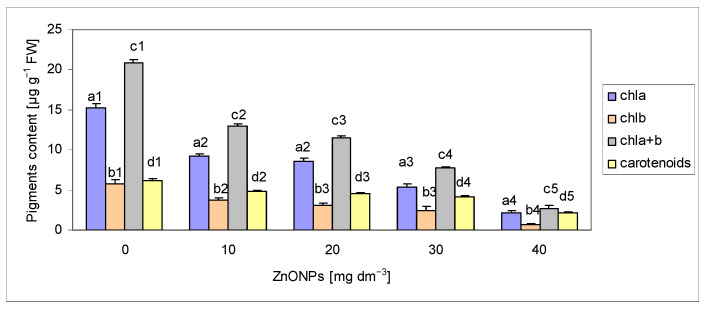
Content of chlorophylls a, b, a + b and carotenoids in fresh weight of *Scutellaria baicalensis* Georgi plantlets. ZnONPs treatments: 0 (control), 10, 20, 30, 40 mg dm^−3^. Error bars represent ± SD. Different letter numbers indicate significant differences at *p* < 0.05.

The contents of chlorophyll a, chlorophyll b, total chlorophyll (a + b), and carotenoids in the fresh biomass of *Scutellaria baicalensis* were statistically significantly lower than in the control group. The reduction in chlorophyll content was greatest at a ZnO nanoparticle concentration of 40 mg dm^−3^. The control value for chlorophyll a was 15.29 μg g^−1^ FW. In samples treated with ZnONPs, the chlorophyll a content decreased with increasing nanoparticle concentration, ranging from 60.8% of the control value at 10 mg dm^−3^ ZnONPs to just 14% at 40 mg dm^−3^ ZnONPs (Figure 4). Chlorophyll b content also declined with increasing ZnONPs concentration, dropping from 65.5% to 10.5% of the control value. The ratio of chlorophyll a to b was 2.6:1. Similarly, the total chlorophyll (a + b) content in the ZnONPs treatments decreased from 62% of the control value at 10 mg dm^−3^ to 13% at 40 mg dm^−3^. The Pearson correlation coefficients between ZnONP concentration and the contents of chlorophyll a, b, and a + b were negative, at r = −0.974, r = −0.976, and r = −0.977, respectively (Table 4). The addition of ZnONPs also led to a reduction in carotenoid content in the fresh biomass of *S. baicalensis*. Statistically significant differences were observed between the control (6.17 μg g^−1^ FW) and plantlets grown in the presence of ZnONPs. Carotenoid content decreased with increasing nanoparticle concentration, from 78% to 34% of the control value (Figure 4). The Pearson correlation coefficient between ZnONPs concentration and carotenoid concentration was r = −0.953 (Table 4).

The highest negative Pearson correlation coefficients were observed between ZnONPs concentrations and the content of photosynthetic pigments. These values ranged from r = −0.953 for carotenoids to r = −0.977 for chlorophyll a + b (Table 4). The positive correlation between ZnONPs concentrations and the total polyphenol content (TPC) and antioxidants measured in extracts from *Scutellaria baicalensis* plantlets using two methods, DPPH and ABTS, was slightly lower, with values of r = 0.631 for TPC, r = 0.625 for DPPH, and r = 0.896 for ABTS. A high positive correlation was found between polyphenol content (TPC) and antioxidant content measured by the DPPH method (r = 0.955). Strong positive correlations were observed between the contents of individual photosynthetic pigments, ranging from r = 0.962 (carotenoids) to r = 0.999 (chlorophyll a + b) (Table 4). Negative correlations between pigment content and antioxidant content measured using the ABTS method were higher (from r = −0.856 to r = −0.909) than for the DPPH method (from r = −0.649 to r = −0.772) (Table 4).

## 3. Discussion

In vitro culture of *Scutellaria baicalensis* was conducted using stem explants with lateral buds and nodes, from which shoots developed through direct organogenesis. Callus often formed at the base of the developing microshoots. Roots regenerated either through callus or directly from the explants. The MS medium supplemented with 1 mg dm^−3^ BAP and 0.1 mg dm^−3^ IBA facilitated effective plant regeneration. In the control group, an average of 10.43 shoots per explant were obtained. Other authors have also carried out micropropagation of *Scutellaria baicalensis*. Kwiecień et al. [27], culturing two species of *Scutellari—S. baicalensis* and *S. lateriflora*, also used MS medium enriched with various combinations of BAP and NAA, ranging from 0.5 to 3 mg dm^−3^. Kawka et al. [28] propagated *S. lateriflora* on MS medium supplemented with various combinations of BAP and NAA under monochromatic, white light, and darkness. The optimal medium for microshoot propagation was found to be MS supplemented with 1–2 mg dm^−3^ BAP and 0.5–1 mg dm^−3^ NAA under blue light. Gharari et al. [29] obtained the highest number of regenerated *Scutellaria bornmuelleri* shoots on MS medium enriched with 1 mg dm^−3^ BAP and 0.5 mg dm^−3^ thidiazuron (TDZ), averaging 24 shoots per explant. A similar composition of MS medium was used for in vitro propagation of *Scutellaria araxensis*; BAP was added alone or in combination with TDZ, indole-3-butyric acid (IBA), or α-naphthaleneacetic acid (NAA). According to the authors, the best direct organogenesis of shoots was obtained using MS medium supplemented with 0.5 mg dm^−3^ BAP and 0.5 mg dm^−3^ IBA (18 shoots per explant) [28,29,30]. Gawroński and Dyduch-Siemińska [15] obtained regenerants of *S. baicalensis* using the method of indirect organogenesis. They observed the most intense shoot organogenesis on MS medium supplemented with kinetin (0.5 mg dm^−3^) and BAP (0.5 mg dm^−3^), which resulted in an average of 7.4 shoots per explant.

In the present study on the micropropagation of *Scutellaria baicalensis*, zinc oxide nanoparticles (ZnONPs) were added to MS medium at concentrations of 10–40 mg dm^−3^. According to the authors’ knowledge, the effect of ZnONPs on the micropropagation of *S. baicalensis* has not previously been studied. The morphological parameters of the regenerated plantlets in the media supplemented with ZnONPs at concentrations of 10–20 mg dm^−3^ did not differ significantly from the control plantlets. Regni et al. [31] observed that ZnONPs at concentrations of 6 and 18 mg dm^−3^ had a beneficial effect on the growth and development of in vitro explants of the olive tree (*Olea europaea* L. cv. “Moraiolo”). Javed et al. [16] reported a positive effect of 1 and 10 mg dm^−3^ ZnONPs on shoot regeneration in another medicinal plant, stevia. In a study by Zafar et al. [17], culturing shoot explants of *Brassica nigra* on MS medium without growth regulators, but with low concentrations of ZnONPs (1–20 mg·dm^−3^), stimulated the regeneration of fine roots with thick root hairs and shoots. In an in vitro culture of *Panicum virgatum*, ZnONPs were used at concentrations of 10–50 mg dm^−3^ to accelerate callus growth. At 10 mg dm^−3^ ZnONPs, a significant increase in callus induction was observed compared to the control [18]. Zinc oxide nanoparticles also accelerated callus growth in *Thymus kotschyanus* and *Zataria multiflora*, increasing secondary metabolite content [32]. ZnONPs may stimulate cell division and proliferation, leading to faster tissue regeneration in plants [33]. In *Solanum tuberosum*, plant growth and regeneration were more rapid when ZnONPs were used. Additionally, ZnONPs had a positive effect on plant growth under salt stress conditions [34]. As shown for various crops, i.e., *Panicum virgatum* L., *Phoenix dactylifera* L., *Coffea arabica* L., and *Solanum lycopersicum* Mill, the concentration of ZnONPs in the medium should be optimized for a given plant species, in order to positively influence callus development, rooting, and somatic embryogenesis [35,36,37,38]. In in vitro cultures of the “Navaho” blackberry variety, ZnONPs at 10 mg dm^−3^ were shown to promote shoot regeneration, while concentrations of 20–40 mg dm^−3^ reduced the number and length of shoots and roots as well as the fresh weight of plantlets [39]. In *Stevia rebaudiana* varieties, a positive effect of ZnONPs on shoot regeneration was observed in vitro concentrations of 30 and 40 mg dm^−3^ for the Morita variety at and at 40 mg dm^−3^ for the Candy variety. However, the highest concentration of 40 mg dm^−3^ ZnONPs in the MS medium negatively affected the number and length of roots, as well as the fresh weight of the plantlets of both varieties [40]. Similarly, in *Scutellaria baicalensis*, high concentrations of zinc oxide nanoparticles limited organogenesis, which affected the increase in fresh plant weight.

For several decades, the global food economy has faced a serious problem of insufficient supply of mineral nutrients, among which zinc is particularly important. The use of ZnONPs in the biofortification of crops with zinc may improve the health benefits of plants, including *Scutellaria baicalensis*. Traditionally cultivated *S. baicalensis* plants are rich in trace elements, but different parts of the plant accumulate minerals in varying amounts. Sheng et al. [41] found that the sequence of six trace elements in the stems of *S. baicalensis* was as follows: Fe > Mn > Zn = Cu > K > Ca. In the leaves, the sequence of six trace elements was similar: Fe > Mn > Zn > Cu > K > Ca. In the flowers and seeds, the order of elements was slightly different, i.e., Ca > Fe > Mn > Zn > Cu > K and Ca > Fe > Zn > Mn > Cu > K, respectively, while in the roots, it was Ca > Fe > Cu > Mn > Zn > K. In our study, the sequence of the elements measured in *S. baicalensis* plantlets from in vitro culture was K > Ca > Mg > Fe > Mn > Zn > Cu. Results reported by Zhu et al. [42] show that the main mineral components in the roots, stems, and leaves of *S. baicalensis* are similar. The ratio of Zn, Mn, Cu, Fe, Mg, Ca, and K content in the leaves was 1:3:6:15:41:333:423. In our study, the ratio of Cu, Zn, Mn, Fe, Mg, Ca, and K content in control plantlets from in vitro culture was 1:19:33:50:231:671:3026. The addition of zinc oxide nanoparticles significantly increased the content of this element in the plant and caused changes in the content of the other elements. Data on the content of inorganic elements in traditionally cultivated *S. baicalensis* plants varies significantly. The mineral content is influenced by soil type, weather conditions, cultivation methods, and fertilization, as well as by the plant parts in which the composition is measured [43]. *S. baicalensis* microshoots from in vitro culture generally have lower content of minerals than mature plants from traditional cultivation [44]. In in vitro culture, the addition of ZnONPs to the medium significantly increased the Zn content in the plants. At the lowest concentration of 10 mg dm^−3^ ZnONPs, the Zn content was higher than that obtained in traditionally grown plants, which ranged from 12.32 to 31.44 mg kg^−1^ [44]. Plants of various *Scutellaria* species are rich in phenolic compounds. Kwiecień et al. [27] analyzed methanol extracts obtained from the biomass of *S. baicalensis* and *S. lateriflora* using HPLC and detected specific flavonoids (baicalin, wogonin, wogonoside, scutellarin, and chrysin), phenylpropanoid glycosides (verbascoside and isoverbascoside), and phenolic acids (p-hydroxybenzoic acid, caffeic acid, ferulic acid, and m-coumaric acid). The extracts exhibited good free radical scavenging activity and moderate reducing power and chelating activity. The content of phenolic compounds in *Scutellaria* plants depends on the species, cultivation conditions, and also the extraction conditions [45,46]. In in vitro culture of *S. lateriflora*, the amounts of flavonoids, phenolic acids, and their precursors varied widely, depending on the presence of growth regulators in the medium and on light conditions [28]. In mature *S. baicalensis* plants from two regions of Ukraine, the total content of polyphenolic compounds ranged from 42.43 to 86.13 mg GAE g^−1^ DW [45]. Mature *S. baicalensis* plants had a total polyphenol (TP) content of 91.83 mg GAE g^−1^ FW, while for other *Scutellaria* species, the TP content in extracts from fresh leaves varied, ranging from 52.11 mg GAE g^−1^ FW for *S. albida* to 281.93 mg GAE g^−1^ FW for *S. ocmulgee* [47]. In the present study, the total content of phenolic compounds in extracts from control plantlets of *Scutellaria baicalensis* was 57 mg GAE g^−1^ FW, and the values were within the range reported for TP by other authors [45,47]. There are many methods for measuring the content of all compounds with antioxidant properties in biological material. Measurements of total antioxidant capacity by the ABTS and DPPH methods showed high antioxidant content in the control plantlets of *Scutellaria baicalensis*; however, the concentrations were lower than in mature, traditionally cultivated plants. In extracts from control plantlets of *Scutellaria baicalensis*, the total antioxidant capacity measured by the ABTS method was 104 µM Trolox g^−1^ FW, while the DPPH method showed 179 µM Trolox g^−1^ FW. Mature *S. baicalensis* plants had a TAC of 1124 µM Trolox g^−1^ FW measured by the ABTS method [47]. *S. altissima* L. plants from in vitro cultures had lower antioxidant levels measured by the ABTS method than field-grown plants, but the phenolic compound content was higher [13,14]. According to Dziurka et al. [48], in vitro *S. baicalensis* and *S. lateriflora* plants contained lower levels of phenolic acids and flavonoids, as well as total extractable antioxidants in methanol extracts, than soil-grown plant material.

ZnO nanoparticles can have a multifaceted impact on plants, including as elicitors of secondary metabolites. In the present study on in vitro culture of *Scutellaria baicalensis*, the total content of phenolic compounds and antioxidant capacity (TAC), measured by the ABTS and DPPH methods, were statistically significantly higher in all samples with ZnONPs than in the control. Zafar et al. [17] reported that concentrations of ZnO nanoparticles ranging from 500 to 1500 mg dm^−3^ negatively affected the seed germination and seedling growth of *Brassica nigra* but led to increased antioxidant activity and phenolic content. The extracts from callus and roots exhibited 79% DPPH scavenging activity at a concentration of 10 mg dm^−3^ ZnONPs. The total antioxidant and reducing potential also significantly depended on the presence of ZnONPs. Depending on the nanoparticle concentration, an increase in polyphenol content (up to 0.15 μg GAE mg^−1^ FW) and flavonoids (up to 0.22 μg QE mg^−1^ FW) was observed [17]. The addition of ZnONPs to the medium in in vitro culture significantly increased the concentration of phenolic compounds and the total antioxidant content in *Stevia rebaudiana* plantlets [40]. In another study, a concentration of 100 mg dm^−3^ ZnONPs resulted in the highest total phenolic content (TPC), total flavonoid content (TFC), and total antioxidant capacity (TAC) in *S. rebaudiana* plantlets [16].

The influence of ZnONPs on the content of secondary metabolites has also been demonstrated in other plant species. The callus of *Echinacea purpurea* obtained from in vitro culture with added ZnONPs contained higher concentrations of flavonoids than the control [19]. Plants increase secondary metabolites or produce them de novo in response to environmental conditions. The plant response to biotic and abiotic stress is associated with the generation of reactive oxygen species (ROS) [49]. Under stress conditions, plants produce reactive oxygen species (ROS), which interact with auxin. This can lead to a reorientation of growth processes as a response to stress. Additionally, auxin itself can induce ROS production. Increasing evidence indicates that the interactions between auxin and reactive oxygen species can modulate plant development in a way that mitigates the effects of environmental stress. Free radicals generated in photosynthesis can influence the action of auxin, e.g., by modifying proteins responsible for auxin transport and regulating the expression of genes regulated by auxin [49,50]. Metal nanoparticles generate free radicals; thus, the stimulation of secondary metabolism by ROS induced by nanoparticles may enhance plant protection against abiotic and biotic stress [22]. The decrease observed in chlorophyll and carotenoid content in the samples with ZnONPs in the present study may indicate a toxic effect of the nanoparticles. In another study, the addition of ZnONPs to an in vitro culture of *Punica granatum* L. affected the content of photosynthetic pigments. Higher total chlorophyll content and higher carotenoid content than the control were recorded at concentrations of 1 and 2.5 mg dm^−3^ ZnO nanoparticles. At 10 mg dm^−3^, however, ZnONPs caused a reduction in their content in pomegranate plants [24]. In contrast, in an in vitro culture of *Salvia officinalis*, the addition of ZnONPs at 10 and 30 mg dm^−3^ significantly increased the total chlorophyll content [23]. Similarly, Regini et al. [51] observed that ZnONPs at concentrations of 6 and 18 mg dm^−3^ exerted a beneficial effect on the content of chlorophyll a and b.

## 4. Materials and Methods

### 4.1. Zinc Oxide Nanoparticles

Zinc oxide nanoparticles (ZnONPs) were sourced from Sigma Aldrich Germany (catalog number 721077, Taufkirchen, Germany). The ZnONPs dispersion was prepared by hydrolysis of a zinc salt in a polyol medium heated to 160°C. According to the manufacturer, the particle size of this product is less than 100 nm based on dynamic light scattering (DLS) measurements, with an average particle size below 35 nm as determined by an aerodynamic particle sizer (APS) spectrometer. In a study by Wang et al. [21], the Zetasizer Nano (Malvern Instruments, Worcestershire, UK) was used to determine that these nanoparticles had an average weighted particle size of 67 ± 2 nm and a zeta potential of +46.1 ± 1.5 mV.

### 4.2. Plant Material

The plant material used in this study was *Scutellaria baicalensis* Georgi, obtained from the experimental farm of the Department of Vegetable and Medicinal Plants at the University of Life Sciences in Lublin, Poland (51°14′53″ N, 2°34′13″ E). In vitro cultures of *S. baicalensis* were initiated on 20 August 2018 at the Laboratory of the Institute of Genetics, Plant Breeding and Biotechnology, University of Life Sciences in Lublin [15]. Shoot apices approximately 3 cm in length, each bearing two or three nodes, were excised from established in vitro cultures and transferred into 200 cm^−3^ glass jars with heat-resistant Magenta B-caps. Each jar contained 20 cm^−3^ of MS (Murashige and Skoog) medium adjusted to pH 5.8, supplemented with 3% sucrose, 0.8% Difco Bacto agar, 1.0 mg·dm^−3^ BAP (6-benzylaminopurine), 0.1 mg·dm^−3^ IBA (indole-3-butyric acid), and ZnONPs at concentrations of 0 (control), 10, 20, 30, and 40 mg·dm^−3^ [52]. The cultures were maintained in a growth chamber at 22–24°C, 60% relative humidity, and a 16 h photoperiod under fluorescent lighting (Fluorescent Lamp Spectral Lux Plus, NL-T8 36W/830/G13, warm white, Radium Lampenwerk GmbH Wipperfürth/Germany) at an intensity of 54 µmol·m^−2^·s^−1^. After three months, the micropropagated plantlets were removed from the culture jars. Measurements were taken for the number and length of shoots and roots and for fresh biomass. The plantlets were subsequently used for further analyses, including determination of mineral content (potassium, calcium, magnesium, iron, zinc, manganese, and copper), photosynthetic pigments (chlorophylls a and b, total chlorophyll a + b, and carotenoids), total phenolic content (TPC), and total antioxidant capacity (TAC). All experiments and analyses were performed in triplicate.

### 4.3. Preparation of Extracts

Plant material was mixed with the extraction solution in a weight-to-volume (*w*/*v*) ratio of 1:10, homogenized for 5 min while cooling in an ice bath, and centrifuged at 5000 rpm for 5 min. The resulting supernatant was frozen at −20°C and used for biochemical analyses. The extraction solvents were as follows: 70% methanol (for phenolic compounds), 80% acetone (for photosynthetic pigments), 70% ethanol (for antioxidant activity using DPPH), and distilled water (for antioxidant activity using ABTS).

### 4.4. Determination of Total Phenolic Content (TPC)

The total phenolic content was determined using the Folin–Ciocalteu spectrophotometric method, which relies on the ability of phenolic compounds to form colored complexes with the reagent’s components (phosphomolybdic and phosphotungstic acid salts) [53].The reaction mixture consisted of 0.025 cm^−3^ of methanolic extract, 0.15 cm^−3^ of Folin–Ciocalteu reagent, 0.3 cm^−3^ of 7.5% sodium carbonate solution, and 2.525 cm^−3^ of distilled water. The absorbance was measured at 765 nm after 1 h of incubation [53]. Results were expressed as gallic acid equivalents (GAE) per 100 g of fresh plant weight.

### 4.5. Determination of Total Antioxidant Capacity

Total antioxidant capacity (TAC) was determined in the extracts using both the ABTS and DPPH methods [54,55]. The ABTS method is based on spectrophotometric measurement of the decolorization reaction between the ABTS⁺ radical cation and antioxidants present in the sample. The colored ABTS⁺ radicals are generated from ABTS (2,2′-azino-bis(3-ethylbenzothiazoline-6-sulfonic acid)) by its reaction with potassium persulfate (K_2_S_2_O_8_). Since ABTS⁺ is soluble in both water and organic solvents, it allows for the assessment of antioxidant activity of hydrophilic compounds. Upon the reduction of ABTS⁺ by antioxidants, the solution loses its color, and the degree of discoloration is proportional to the antioxidant concentration. Absorbance was measured spectrophotometrically at 414 nm, 30 min after the reagents were mixed [54]. The DPPH method uses the stable free radical DPPH (2,2-diphenyl-1-picrylhydrazyl), which appears as a dark violet solution. In the presence of antioxidants, DPPH^·^ is reduced, resulting in a color change and a decrease in absorbance. The reduction in absorbance is directly related to the antioxidant capacity of the sample. Measurements were taken at 517 nm, 30 min after the reagents were mixed [55].

### 4.6. Determination of Mineral Composition

The micropropagated plantlet samples were rinsed with distilled water and dried in a forced-air oven at 70°C for 72 h. The dried plant material was digested using a mixture of nitric acid (HNO_3_) and perchloric acid (HClO_4_) [56]. After appropriate dilution, the digestates were analyzed for the content of potassium (K), calcium (Ca), magnesium (Mg), iron (Fe), manganese (Mn), zinc (Zn), and copper (Cu).

### 4.7. Statistical Analysis

The results were analyzed by analysis of variance (ANOVA), and significant differences between means were evaluated using Tukey’s HSD post hoc test at *p* < 0.05. Pearson correlation coefficients (r) were also calculated to assess the strength of linear relationships between parameters. All statistical analyses were performed using Statistica software version 13.1.

## 5. Conclusions

In this study, a clear effect of ZnO nanoparticles (ZnONPs) on the morphological and physiological characteristics of *Scutellaria baicalensis* under in vitro conditions was observed. Overall, compared to the control plantlets, those regenerated on media supplemented with ZnONPs had higher levels of mineral nutrients and phytocompounds with antioxidant properties, but lower levels of photosynthetic pigments. Within the tested concentration range of 10–40 mg·dm^−3^, ZnONPs had a variable impact on growth parameters and the content of key biologically active compounds, including secondary metabolites and photosynthetic pigments. ZnONPs concentrations of 10 and 20 mg·dm^−3^ did not significantly affect shoot or root regeneration or fresh biomass accumulation; however, they enhanced the accumulation of selected mineral elements, polyphenols, and antioxidants, while reducing levels of photosynthetic pigments. Higher concentrations (30 and 40 mg·dm^−3^) exerted toxic effects, which manifested as reduced organogenesis of *S. baicalensis* explants and a significant decline in chlorophyll and carotenoid content, but at the same time promoted the accumulation of phenolic compounds and antioxidants. These findings suggest that incorporating ZnONPs into micropropagation protocols for *S. baicalensis* Georgi may support more efficient and targeted propagation of this economically important medicinal species. Carefully optimizing ZnONPs concentrations could improve in vitro growth and development, enabling the large-scale production of healthy and vigorous plantlets and ultimately improving economic returns through sustainable and accelerated cultivation practices.

## Figures and Tables

**Table 1:** Effect of increasing ZnONPs concentration in MS solid medium on the biometric characteristics of *Scutellaria baicalensis* Georgi plantlets after three months of in vitro culture (mean values per explant).

Biometric Feature	Concentration of ZnONPs [mg dm^−3^]					
	0 (Control)	10	20	30	40	LSD *p* < 0.05
Number of shoots	10.43 ^a^*	8.94 ^a^	8.77 ^ab^	5.76^bc^	2.92 ^c^	3.585
Shoot length [cm]	8.91 ^a^	8.58 ^a^	7.63 ^ab^	5.83 ^bc^	3.05 ^c^	2.905
Number of roots	4.19^a^	3.86^ab^	2.79 ^bc^	1.92 ^bc^	1.55 ^c^	1.739
Root length [cm]	3.64 ^a^	3.06 ^a^	2.25 ^ab^	1.80 ^b^	0.81 ^b^	1.705
Fresh weight of plant [g] (Increment [g])	0.686 ^a^ (0.657) ^a^	0.702 ^a^ (0.681) ^a^	0.658 ^a^ (0.632) ^a^	0.418 ^b^ (0.394) ^b^	0.109 ^b^ (0.092) ^b^	0.255 (0.232)

* Means with the same superscript letter within a row are not significantly different according to Tukey’s test (*p* < 0.05).

**Table 2:** Effect of ZnONPs on mineral content in plantlets of *Scutellaria baicalensis* Georgi after three months of in vitro culture.

ZnONPs [mg dm^−3^]	Mineral Content [mg 100 g^−1^ DW]						
	K	Ca	Mg	Fe	Mn	Zn	Cu
0	2330 a	517 a	178 a	38.7 a	25.4 a	14.6 a	0.77 a
10	2455 b	552 b	195 b	43.5 b	25.9 a	42.3 b	0.75 a
20	2520 c	582 c	203 c	56.2 c	26.0 a	69.2 c	0.73 ab
30	3440 d	507 d	219 d	33.1 d	22.7 b	211.0 d	0.69 b
40	2260 e	453 e	175 a	32.5 d	21.2 b	236.0 e	0.64 b
LSD *p* < 0.05	13.50	9.15	7.86	2.11	3.55	6.30	0.09

Means with the same letter within columns are not significantly different according to Tukey’s test (*p* < 0.05).

**Table 3:** Effect of ZnO nanoparticles on mineral content proportions in plantlets of *Scutellaria baicalensis* Georgi after three months of in vitro culture.

ZnONPs [mg dm^−3^]	Mineral Content Ratio						
	K	Ca	Mg	Fe	Mn	Zn	Cu
0	3026	671	231	50	33	19	1
10	3273	736	260	58	35	56	1
20	3452	797	278	77	36	95	1
30	4986	735	317	48	33	306	1
40	3531	708	273	51	33	369	1

Cu content was assigned a value of 1.

**Table 4:** Pearson’s correlation coefficients (r) between measurements.

Measurements	ZnONPs Treatments	TPC	DPPH	ABTS	Chl a	Chl b	Chl a + b	Carotenoids
ZnONPs treatments	1							
TPC	0.631	1						
DPPH	0.625	0.955	1					
ABTS	0.896	0.690	0.565	1				
chl a	−0.974	−0.756	−0.772	−0.856	1			
chl b	−0.976	−0.744	−0.722	−0.909	0.989	1		
chl a + b	−0.977	−0.754	−0.760	−0.872	0.999	0.994	1	
carotenoids	−0.953	−0.675	−0.649	−0.883	0.962	0.986	0.971	1

Correlation coefficients are significant at *p* < 0.05.

## Data Availability

The original contributions presented in this study are included in the article. Further inquiries can be directed to the corresponding author.

## References

[1] Bochoráková H., Paulová H., Slanina J., Musil P., Táborská E. (2003). Main flavonoids in the root of *Scutellaria baicalensis* cultivated in Europe and their comparative antiradical properties. Phytother. Res..

[2] Zhao Q., Yang J., Cui M.Y., Liu J., Fang Y.M., Yan M.X., Qiu W.Q., Shang H.W., Xu Z.C., Yidiresi R. (2019). The reference genome sequence of *Scutellaria baicalensis* provides insights into the evolution of wogonin biosynthesis. Mol. Plant.

[3] Zhao T., Tang H., Xie L., Zheng Y., Ma Z., Sun Q., Li X. (2019). *Scutellaria baicalensis* Georgi. (Lamiaceae): A review of its traditional uses, botany, phytochemistry, pharmacology and toxicology. J. Pharm. Pharmacol..

[4] Spyrka K., Brela J. (2023). *Scutellaria baicalensis* as new hydrogel formulation with antioxdant propertie. Forum Leczenia Ran.

[5] Cai J., Hu Q., He Z., Chen X., Wang J., Yin X., Ma X., Zeng J. (2023). *Scutellaria baicalensis* Georgi and their natural flavonoid compounds in the treatment of ovarian cancer: A review. Molecules.

[6] Tan Y.Q., Lin F., Ding Y.K., Dai S., Liang Y.X., Zhang Y.S., Li J., Chen H.W. (2022). Pharmacological properties of total flavonoids in *Scutellaria baicalensis* for the treatment of cardiovascular diseases. Phytomedicine.

[7] Wang J., Chen S., Zhang J., Wu J. (2022). *Scutellaria baicalensis* Georgi is a promising candidate for the treatment of autoimmune diseases. Front. Pharmacol..

[8] Ozma M.A., Khodadadi E., Pakdel F., Kamounah F.S., Yousefi M., Yousefi B., Asgharzadeh M., Ganbarov K., Kafil H.S. (2021). Baicalin, a natural antimicrobial and antibiofilm agent. J. Herb. Med..

[9] Song J.W., Long J.Y., Xie L., Zhang L.L., Xie Q.X., Chen H.J., Deng M., Li X.F. (2020). Applications; phytochemistry; pharmacological effects; pharmacokinetics; toxicity of *Scutellaria baicalensis* Georgi. and its probably potential therapeutic effects on COVID-19: A review. Chin. Med..

[10] Yin B., Li W., Qin H., Yun J., Sun X. (2021). The use of Chinese skullcap (*Scutellaria baicalensis*) and its extracts for sustainable animal production. Animals.

[11] Li Y., Xie X., Wang C., Pei X., Fang C., Yan R., Zhong Y. (2024). Antibacterial and UV resistant functions of cotton fabrics: In- situ free radical polymerization of AgNPs using Baicalin. Ind. Crops Prod..

[12] Ordon M., Nawrotek P., Stachurska X., Schmidt A., Mizielińska M. (2021). Mixtures of *Scutellaria baicalensis* and *Glycyrrhiza* L. extracts as antibacterial and antiviral agents in active coatings. Coatings.

[13] Grzegorczyk-Karolak I., Kuźma Ł., Wysokińska H. (2016). In vitro cultures of *Scutellaria alpina* as a source of pharmacologically active metabolites. Acta Physiol. Plant..

[14] Grzegorczyk-Karolak I., Kuźma Ł., Wysokińska H. (2015). Study on the chemical composition and antioxidant activity of extracts from shoot culture and regenerated plants of *Scutellaria altissima* L.. Acta Physiol. Plant..

[15] Gawroński J., Dyduch-Siemińska M. (2022). Potential of in vitro culture of *Scutellaria baicalensis* in the formation of genetic variation confirmed by ScoT Markers. Genes.

[16] Javed R., Yucesan B., Zia M., Ekrem G. (2018). Elicitation of secondary metabolites in callus cultures of *Stevia rebaudiana* Bertoni grown under ZnO and CuO nanoparticles stress. Sugar Tech..

[17] Zafar H., Ali A.A., Ali J.S., Haq I.U., Zia M. (2016). Effect of ZnO nanoparticles on *Brassica nigra* seedlings and stem explants: Growth dynamics and antioxidative response. Front. Plant Sci..

[18] Tymoszuk A., Wojnarowicz J. (2020). Zinc oxide and zinc oxide nanoparticles impact on in vitro germination and seedling growth in *Allium cepa* L.. Materials.

[19] Karimi N., Behbahani M., Dini G., Razmjou A. (2018). Enhancing the secondary metabolite and anticancer activity of *Echinacea purpurea* callus extracts by treatment with biosynthesized ZnO nanoparticles. Adv. Nat. Sci. Nanosci. Nanotechnol..

[20] Taheri M., Mousavi M., Mortazavi S.M.H. (2024). Different reactions of olive explants in response to zinc oxide nanoparticles and zinc sulfate under in vitro conditions. Int. J. Hortic. Sci..

[21] Wang P., Menzies N.W., Lombi E., McKenna B.A., Johannessen B., Glover C.J., Kappen P., Kopittke P.M. (2013). Fate of ZnO nanoparticles in soils and cowpea (*Vigna unguiculata*). Environ. Sci. Technol..

[22] Hassan M.U., Aamer M., Chattha M.U., Haiying T., Shahzad B., Barbanti L., Nawaz M., Rasheed A., Afzal A., Liu Y. (2020). The critical role of zinc in plants facing the drought stress. Agriculture.

[23] Alenezi N.A., Al-Qurainy F., Tarroum M., Nadeem M., Khan S., Salih A.M., Shaikhaldein H.O., Alfarraj N.S., Gaafar A.-R.Z., Al-Hashimi A. (2022). Zinc oxide nanoparticles (ZnO NPs); biosynthesis; characterization and evaluation of their impact to improve shoot growth and to reduce salt toxicity on *Salvia officinalis* in vitro cultivated. Processes.

[24] El-Mahdy M.T., Elazab D.S. (2020). Impact of zinc oxide nanoparticles on pomegranate growth under in vitro conditions. Russ. J. Plant Physiol..

[25] Rossi L., Fedenia L.N., Sharifan H., Ma X., Lombardini L. (2019). Effects of foliar application of zinc sulfate and zinc nanoparticles in coffee (*Coffea arabica* L.) Plants. Plant Physiol. Biochem..

[26] Subba B., Rai G.B., Bhandary R., Parajuli P., Thapa N., Kandel D.R., Mulmi S., Shrestha S., Malla S. (2024). Antifungal activity of zinc oxide nanoparticles (ZnO NPs) on *Fusarium equiseti* phytopathogen isolated from tomato plant in Nepal. Helyon.

[27] Kwiecień I., Łukaszyk A., Miceli N., Tawiano M.F., Davi F., Kędzia E., Ekier H. (2023). In vitro cultures of *Scutellaria brevibracteata* subsp. *subvelutina* as a source of bioactive phenolic metabolites. Molecules.

[28] Kawka B., Kwiecień I., Ekiert H. (2017). Influence of culture medium composition and light conditions on the accumulation of bioactive compounds in shoot cultures of *Scutellaria lateriflora* L. (american skullcap) grown in vitro. Appl. Biochem. Biotechnol..

[29] Gharari Z., Bagheri K., Karimkhanlooei G., Sharafi A. (2021). Study of tissue culture and in vitro organogenesis of *Scutellaria bornmuelleri* using benzylaminopurine; lsopentenyl adenine and thidiazuron. S. Afr. J. Bot..

[30] Gharari Z., Bagheri K., Sharafi A. (2022). High-frequency adventitious shoot organogenesis from in vitro stem explants of *Scutellaria araxensis* Grossh. BioTechnologia.

[31] Regni L., Del Buono D., Micheli M., Facchin S.L., Tolisano C., Proietti P. (2022). Effects of biogenic ZnO nanoparticles on growth; physiological; biochemical traits and antioxidants on olive tree in vitro. Horticulturae.

[32] Mosavat N., Golkar P., Yousefifard M., Javed R. (2019). Modulation of callus growth and secondary metabolites in different *Thymus* species and *Zataria multiflora* micropropagated under ZnO nanoparticles stress. Biotechnol. Appl. Biochem..

[33] Geremew A., Carson L., Woldesenbet S., Wang H., Reeves S., Brooks N., Saganti P., Weerasooriya A. (2023). Effect of zinc oxide nanoparticles synthesized from *Carya illinoinensis* leaf extract on growth and antioxidant properties of mustard (*Brassica juncea*). Front. Plant Sci..

[34] Alghamdi E.S., Farooq M., Metwali E.M.R. (2022). Influence of nano zinc oxide on the in vitro callus growth; ex vitro tuber yield and nutrional quality of potato (*Solanum tuberosum* L.) cultivars under salt stress. J. Anim. Plant Sci..

[35] Alharby H.F., Metwali E.M.R., Fuller M.P., Aldhebiani A.Y. (2016). Impact of application of zinc oxide nanoparticles on callus induction; plant regeneration; element content; and antioxidant enzyme activity in tomato (*Solanum lycopersicum* Mill.) under salt stress. Acta Sci. Biol. Sci..

[36] Devasia J., Muniswamy B., Mishra M.K. (2020). Investigation of ZnO nanoparticles on in vitro cultures of coffee (*Coffea arabica* L.). Int. J. Nanosci. Nanotechnol..

[37] Shafique N., Jabeen K.S., Ahmad S., Irum S., Anwaar N., Ahmad S., Alam M., Ilyas T.F., Khan S.Z., Hussain S. (2020). Green fabricated zinc oxide nanoformulated media enhanced callus induction and regeneration dynamics of *Panicum virgatum* L.. PLoS ONE.

[38] Al-Mayahi A.M.W. (2021). The effect of humic acid (HA) and zinc oxide nanoparticles (ZnO-NPs) on in vitro regeneration of date palm (*Phoenix dactylifera* L.) cv. Quntar. Plant Cell Tissue Organ Cult..

[39] Krzepiłko A., Prażak R., Matyszczuk K. (2024). Influence of zinc oxide nanoparticles in in vitro culture and bacteria *Bacillus thuringiensis* in *ex vitro* conditions on the growth and development of blackberry (*Rubus fruticosus* L.). Appl. Sci..

[40] Krzepiłko A., Prażak R., Matyszczuk K., Dyduch-Siemińska M. (2024). The effect of zinc oxide nanoparticles on the growth and development of *Stevia* plants cultured in vitro. Acta Sci. Pol. Hortorum Cultus.

[41] Sheng J.P., Chen H.R., Shen L. (2009). Determination of six mineral elements in roots; stems; leaves; flowers and seeds of *Scutellaria baicalensis* by FAAS. Spectrosc. Spectr. Anal..

[42] Zhu Y.-X., Luo X., Zhao D.-P., Zhai Z.-X., Guo Y.-H. (2011). Analysis of the content of mineral elements in *Scutellaria baicalensis*; skullcap tea and its solution. Spectrosc. Spectr. Anal..

[43] Guo L., Wang S., Zhang J., Yang G., Zhao M., Ma W., Zhang X., Li X., Han B., Chen N. (2013). Effects of ecological factors on secondary metabolites and inorganic elements of *Scutellaria baicalensis* and analysis of geoherblism. Sci. China Life Sci..

[44] Li X., Wei X.-F., Wu J., Yin Z.-Q., Wan L.-Q., Sun H.-Y., An Y.-L. (2022). Geochemical characteristics and growth suitability assessment of *Scutellaria baicalensis* Georgi in the Earth’s critical zone of North China. J. Mt. Sci..

[45] Vergun O., Svydenko L., Grygorieva O., Shymanska O., Rakhmetov D., Brindza J., Ivanišová E. (2019). Antioxidant capacity of plant raw material of *Scutellaria baicalensis* Georgi. Potravin. Slovak J. Food Sci..

[46] Lim J., Kim K., Kwon D.Y., Kim J.K., Sathasivam R., Park S.U. (2024). Effects of different solvents on the extraction of phenolic and flavonoid compounds; and antioxidant activities; in *Scutellaria baicalensis* hairy roots. Horticulturae.

[47] Vaidya B.N., Brearley T.A., Joshee N. (2014). Antioxidant capacity of fresh and dry leaf extracts of sixteen *Scutellaria* species. J. Med. Act. Plants.

[48] Dziurka M., Kubica P., Kwiecień I., Biesaga-Kościelniak J., Ekiert H., Abdelmohsen S.A.M., Al-Harbi F.F., El-Ansary D.O., Elansary H.O., Szopa A. (2021). In vitro cultures of some medicinal plant species (*Cistus* × *incanus*; *Verbena officinalis*; *Scutellaria* lateriflora; and *Scutellaria baicalensis*) as a rich potential source of antioxidants-evaluation by cuprac and quencher-cuprac assays. Plants.

[49] Sachdev S., Ansari S.A., Ansari M.I., Fujita M., Hasanuzzaman M. (2021). Abiotic stress and re-active oxygen species: Generation; signaling; and defense mechanisms. Antioxidants.

[50] Parveen N., Kandhol N., Sharma S., Singh V.P., Chauhan D.K., Ludwig-Müller J., Tripathi D.K. (2022). Auxin crosstalk with reactive oxygen and nitrogen species in plant development and abiotic stress. Plant Cell Physiol..

[51] Regni L., Del Buono D., Micheli M., Facchin S.L., Cesarini A., Priolo D., Proietti P. (2024). Biogenic zinc oxide nanoparticles improve in vitro growth of blueberries. Horticulturae.

[52] Murashige T., Skoog F. (1962). A revised medium for rapid growth and bio assays with tobacco tissue cultures. Physiol. Plant..

[53] Singleton V.L., Orthofer R., Lamuela-Raventos R.M. (1999). Analysis of total phenols and other oxidation substrates and antioxidants by means of Folin-Ciocalteu reagent. Meth. Enzymol..

[54] Re R., Pellegrini N., Proteggente A., Pannala A., Yang M., Rice-Evans C. (1999). Antioxidant activity applying an improved ABTS radical cation decolorization assay. Free Radic. Biol. Med..

[55] Addai Z.R., Abdullah A., Mutalib S.A. (2013). Effect of extraction solvents on the phenolic content and antioxidant properties of two papaya cultivars. J. Med. Plants Res..

[56] (2002). Forage. Determine the Content of Calcium; Copper; Iron; Magnesium; Manganese and Zinc-Method by Atomic Absorption Spectrometry.

